# Tacrolimus- and Mycophenolate-Mediated Toxicity: Clinical Considerations and Options in Management of Post-Transplant Patients

**DOI:** 10.3390/cimb47010002

**Published:** 2024-12-24

**Authors:** Alan D. Kaye, Shivam S. Shah, Coplen D. Johnson, Adalyn S. De Witt, Austin S. Thomassen, Charles P. Daniel, Shahab Ahmadzadeh, Sridhar Tirumala, Kristin Nicole Bembenick, Adam M. Kaye, Sahar Shekoohi

**Affiliations:** 1 Departments of Anesthesiology and Pharmacology, Toxicology, and Neurosciences, Louisiana State University Health Sciences Center Shreveport, Shreveport, LA 71103, USA; 2 School of Medicine, Louisiana State University Health Sciences Center at Shreveport, Shreveport, LA 71103, USA; sss002@lsuhs.edu (S.S.S.); cdj002@lsuhs.edu (C.D.J.); cpd002@lsuhs.edu (C.P.D.); 3 School of Medicine, Indiana University, 340 W 10th St., Indianapolis, IN 46202, USA; 4 Department of Anesthesiology, Louisiana State University Health Sciences Center Shreveport, Shreveport, LA 71103, USA; 5 Department of Pharmacy Practice, Thomas J. Long School of Pharmacy, University of the Pacific, 751 Brookside Road, Stockton, CA 95207, USA

**Keywords:** tacrolimus, mycophenolate, post-transplant, toxicity, immunosuppression

## Abstract

Tacrolimus and mycophenolate are important immunosuppressive agents used to prevent organ rejection in post-transplant patients. While highly effective, their use is associated with significant toxicity, requiring careful management. Tacrolimus, a calcineurin inhibitor, is linked to nephrotoxicity, neurotoxicity, metabolic disturbances such as diabetes mellitus and dyslipidemia, and cardiovascular complications such as hypertension and arrhythmias. Mycophenolate, a reversible inhibitor of inosine monophosphate dehydrogenase, frequently causes gastrointestinal disturbances, including diarrhea and colitis, as well as hematologic side effects like anemia and leukopenia, which increase infection risk. Therapeutic drug monitoring (TDM) and pharmacogenomics have emerged as essential strategies for mitigating these toxicities. TDM ensures tacrolimus trough levels are maintained within a therapeutic range, minimizing the risks of nephrotoxicity and rejection. Pharmacogenomic insights, such as CYP3A5 polymorphisms, allow for personalized tacrolimus dosing based on individual metabolic profiles. For mycophenolate, monitoring inosine monophosphate dehydrogenase activity provides a pharmacodynamic approach to dose optimization, reducing gastrointestinal and hematologic toxicities. Emerging tools, including dried blood spot sampling and pharmacokinetic modeling, offer innovative methods to simplify monitoring and enhance precision in outpatient settings. Despite their utility, the toxicity profiles of these drugs, including those of early immunosuppressants such as cyclosporine and azathioprine, necessitate further consideration of alternative immunosuppressants like sirolimus, everolimus, and belatacept. Although promising, these newer agents require careful patient selection and further research. Future directions in immunosuppressive therapy include integrating individual pharmacogenetic data to refine dosing, minimize side effects, and improve long-term graft outcomes. This narrative review underscores the importance of personalized medicine and advanced monitoring in optimizing post-transplant care.

## 1. Introduction

Tacrolimus and mycophenolate mofetil are commonly used immunosuppressive agents, being primarily indicated to prevent acute organ transplant rejection and occasionally used for autoimmune conditions [1,2]. Each drug has distinct mechanisms of action, pharmacokinetics, and side-effect profiles, allowing for their use either alone or in combination, depending on clinical needs. Tacrolimus, a calcineurin inhibitor, is mainly used in kidney, liver, and heart transplants. It has shown advantages over cyclosporine, another calcineurin inhibitor, by providing improved long-term graft survival in kidney and liver transplants [3,4]. Although tacrolimus is nephrotoxic, it is less so than cyclosporine, making careful dose management and renal monitoring crucial [5]. Mycophenolate mofetil, on the other hand, is often used in combination with other immunosuppressants related to its ability to inhibit lymphocyte proliferation effectively while preserving kidney function, an advantage over more nephrotoxic drugs [6].

Post-transplant immunosuppression is essential for graft survival, as it prevents the recipient’s immune system from recognizing and attacking the transplanted organ as foreign [7]. Immunosuppressive drugs like tacrolimus and mycophenolate mofetil work by downregulating the immune response to prevent acute rejection, a common and potentially severe complication [8]. Hyperacute and chronic rejection are other complications of organ transplant; however, immunosuppressive therapy mainly seeks to modulate acute rejection [9]. Acute rejection is an immune-mediated response that can arise within weeks to months after transplantation or later if immunosuppression is insufficient [10]. T-cell-mediated rejection, the most common form, occurs when recipient T cells recognize donor antigens as foreign, leading to inflammation and graft damage [11]. Symptoms vary by organ; for instance, kidney rejection may manifest as increased serum creatinine, reduced urine output, and tenderness, while liver rejection may cause elevated liver enzymes, jaundice, and fatigue [9,12]. Immunosuppressive regimens must be carefully managed to prevent rejection while minimizing infection risks, as immune suppression increases susceptibility to infections (viral, bacterial, and fungal) and certain malignancies [13]. Striking the right balance in immunosuppressive therapy optimizes graft survival and ensures a manageable infection risk for the patient.

**Figure 1:**
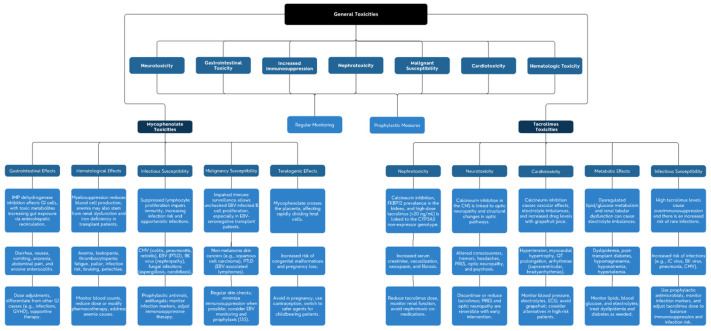
Comprehensive summary of general toxicities, tacrolimus-specific and mycophenolate-specific toxicities, and mitigation strategies.

This narrative review, therefore, aims to introduce the roles of tacrolimus and mycophenolate mofetil in transplant medicine, with a focus on their pharmacokinetics, pharmacodynamics, and toxicity profiles. It will explore how these toxicities impact patients, examine drug–drug interactions between tacrolimus, mycophenolate mofetil, and other immunosuppressants, and discuss strategies for minimizing adverse effects. Finally, we will highlight emerging therapies and novel immunosuppressive agents that may offer reduced side-effect profiles, enhancing patient outcomes and graft longevity in the field of transplant medicine. Figure 1 provides a comprehensive visual summary of the general toxicities, drug-specific effects, and mitigation strategies for tacrolimus and mycophenolate.

## 2. Pharmacokinetics and Pharmacodynamics of Tacrolimus and Mycophenolate

Tacrolimus binds to FK506-binding protein, forming a complex that inhibits calcineurin, a calcium/calmodulin-dependent serine–threonine phosphatase essential for activating nuclear factor of activated T cells (NFAT) [14]. Calcineurin achieves this by dephosphorylating NFAT, allowing it to enter the nucleus [15]. Once inside, NFAT promotes the transcription of interleukin-2 (IL-2), a key cytokine that stimulates cytotoxic CD8+ T cells and natural killer (NK) cells, both of which are involved in acute transplant rejection [16]. By inhibiting calcineurin, tacrolimus indirectly suppresses IL-2 production, thereby reducing T-cell-mediated responses against transplanted organs, although it also results in a weakened immune system [17].

Mycophenolate mofetil is a reversible inhibitor of inosine monophosphate (IMP) dehydrogenase, the enzyme that converts IMP into xanthosine monophosphate (XMP), a precursor for guanosine monophosphate (GMP) synthesis [18]. GMP is a vital purine nucleotide required for DNA synthesis in rapidly dividing cells like T and B lymphocytes [19]. By inhibiting IMP dehydrogenase, mycophenolate mofetil disrupts lymphocyte proliferation, effectively helping to prevent acute transplant rejection, although it also suppresses immune function [20].

With regard to pharmacokinetics, tacrolimus is variably absorbed in the gastrointestinal tract, with a bioavailability of 11.2–19.1% in one study [21]. It is highly lipophilic and extensively binds to plasma proteins, such as albumin, resulting in a high volume of distribution and good tissue penetration, especially in the liver and kidneys [22]. Tacrolimus is primarily metabolized by the cytochrome P450 enzymes CYP3A4 and CYP3A5, leading to significant drug–drug interactions, and it is mainly excreted in bile [23,24]. Variability in hepatic function can affect tacrolimus’s half-life, which is typically 12 h in adults [25].

Mycophenolate mofetil, a prodrug, converts to its active form, mycophenolic acid (MPA), upon absorption, with a high bioavailability of approximately 94% [26]. MPA binds to plasma proteins, limiting its free fraction and ability to exert therapeutic effects [27]. Widely distributed to tissues, MPA is metabolized in the liver to the inactive mycophenolic acid glucuronide (MPAG) through glucuronidation [28]. MPAG undergoes enterohepatic recirculation, resulting in a secondary peak in plasma levels about 6–12 h post-administration, and is excreted via the kidneys with a half-life of 9–17 h [29,30]. Both drugs require careful monitoring, as their absorption, metabolism, and elimination are influenced by other medications and individual patient factors.

## 3. General Toxicity of Immunosuppressants in Post-Transplant Patients

Immunosuppressant-related toxicity is a significant concern in transplant patients, as these medications, while crucial for preventing organ rejection, can have adverse effects on various organ systems. Nephrotoxicity is common with drugs like calcineurin inhibitors (e.g., tacrolimus and cyclosporine), which can cause kidney damage over time, potentially leading to chronic kidney disease in patients with heart, lung, or liver transplants [31]. Infections are also a major risk, as immunosuppressive therapy, including tacrolimus, weakens the immune system, increasing susceptibility to bacterial, viral, protozoal, and fungal infections, particularly during the early post-transplant period when higher doses are used [32]. This immunosuppression also heightens the risk of opportunistic infections, including cytomegalovirus (CMV) and Epstein–Barr virus (EBV), as well as certain cancers like skin cancer and post-transplant lymphoproliferative disorder (PTLD) [33,34]. Additionally, all immunosuppressants, including tacrolimus, elevate the risk of cancers, especially skin malignancies, and developing lymphomas [35]. This risk is more closely associated with the intensity and duration of immunosuppression than with the specific medication used.

Beyond infections and cancer risk, immunosuppressants can lead to cardiovascular toxicity. Corticosteroids, cyclosporine, and tacrolimus can elevate blood pressure, cholesterol, and blood glucose levels, increasing the risk of cardiovascular disease [36]. Hematologic toxicity can also occur, with drugs like mycophenolate mofetil potentially causing bone marrow suppression, leading to anemia, leukopenia, or thrombocytopenia [37,38]. Gastrointestinal toxicity is common as well, with mycophenolate mofetil frequently causing GI symptoms like nausea, vomiting, diarrhea, and gastritis, which may limit its tolerability in some patients [39].

Additionally, neurotoxicity is a notable side effect with calcineurin inhibitors, which can lead to tremors, headache, confusion, and, in severe cases, seizures [40]. Hepatotoxicity may also occur with some immunosuppressants, especially azathioprine and tacrolimus, which can affect liver function and necessitate close monitoring of liver enzymes [41,42]. Managing these toxicities requires careful monitoring, dose adjustments, and sometimes switching medications to achieve a balance between preventing organ rejection and minimizing adverse effects.

Tacrolimus and mycophenolate mofetil share several common adverse effects, primarily due to their immunosuppressive action. Both increase the susceptibility to infections, including bacterial, viral, and fungal infections, as well as certain opportunistic infections [43,44]. Gastrointestinal symptoms are also common, especially with mycophenolate mofetil [39]. Both drugs can lead to hematologic toxicity, causing leukopenia and anemia, leading to fatigue [45]. Additionally, long-term use of these drugs enhances the risk of cancers, such as skin cancers and PTLD, due to weakened immune surveillance [46,47]. Regular monitoring and dose adjustments are important to minimize these toxicities while effectively preventing organ rejection.

## 4. Tacrolimus-Related Toxicity in Post-Transplant Patients

Tacrolimus, a key part of post-transplant immunosuppressive therapy, is associated with a wide range of toxicities that impact multiple organ systems, requiring careful monitoring and management to mitigate risks (Table 1). For over two decades, tacrolimus nephrotoxicity has been a well-documented side effect in post-transplant patients. Tacrolimus exerts its immunosuppressive effects via calcineurin inhibition by binding to cyclophilin A and FK506 binding protein 12 (FKBP12) [48], which is also implicated in its nephrotoxic properties. It has been postulated that calcineurin inhibition is also related to the mechanism for nephrotoxicity [48]. The incidence of nephrotoxicity ranges from 17 to 44% in renal transplant patients and 18 to 42% in liver transplant patients [48]. There is a high prevalence of FKBP12 in the kidney in relation to the liver and spleen, which may be an indicator of increased nephrotoxicity in tacrolimus users compared to hepatotoxicity [48].

Renal biopsies in tacrolimus-treated patients often reveal distinct morphological changes, including epithelial vacuolization, vasospasm, and interstitial fibrosis [48]. These changes present with an increase in blood urea or serum creatinine levels and are isolated from other causes of the clinical presentation [48]. A diagnosis of tacrolimus nephrotoxicity is usually made via exclusion and supported by a decrease in serum creatinine after the tacrolimus dosage is decreased [48]. It has been found that tacrolimus nephrotoxicity is correlated to the dosage of tacrolimus that the patient is receiving [49]. Prescribing the proper dose of tacrolimus is vital, as the toxicity threshold has been seen to be 20 ng/mL^2^ [49]. Although tacrolimus dosage is critical, other factors, such as the donor’s CYP3A5 non-expressor genotype, increase the likelihood of calcineurin inhibitor (tacrolimus) nephrotoxicity [50]. This is a logical effect, as the cytochrome P450 system metabolizes tacrolimus and determines its serum concentration.

Beyond nephrotoxicity, tacrolimus is associated with significant neurotoxicity, including symptoms like altered consciousness, tremors, headaches, posterior reversible encephalopathy syndrome, optic neuropathy, and psychosis [51,52,53,54]. Posterior reversible leukoencephalopathy syndrome has a variable presentation that may include white matter lesions affecting the parieto-occipital lobes and is documented to be the most common neurotoxic effect of tacrolimus [55,56]. This displays clinically as seizures, confusion, and altered vision [55,56]. In the event of cortical blindness, the reversal of symptoms was found in a case study with the discontinuation of tacrolimus [55]. Optic neuropathy is a rare side effect of tacrolimus that exhibits decreased visual acuity, changes in color vision, pupillary defects, and decreased visual fields [54]. Optic neuropathy may also present with hyperintensities along the optic nerves, optic chiasm, and anterior brainstem [54]. Case reports document that neurotoxicity including optic neuropathy is reversible with discontinuation of tacrolimus [57]. However, it is important to consider the systemic effects of discontinuation considering the vital importance of tacrolimus in the immunosuppressive regimen of transplant patients.

Among the varied toxicities of tacrolimus are cardiovascular toxicities. There are many etiologies of hypertension, including age, nervous system effects, renin–angiotensin–aldosterone system changes, the upregulation of peptides, kidney transplant dysfunction or rejection, renal artery stenosis, and others, in addition to drug-related toxicity [58,59]. In the case of tacrolimus, calcineurin inhibition can potentiate hypertension, particularly as it relates to vascular and neural causes of hypertension [60]. There are drug-mediated effects of tacrolimus that increase the likelihood of hypertension, including salt reabsorption in the tubules, vasoconstriction, and sympathetic nervous system activation [58]. Rarely, tacrolimus has also been associated with causing drug-induced myocardial hypertrophy [61]. Higher blood levels of tacrolimus were linked to a greater risk of developing myocardial hypertrophy; however, this was found to be reversible. While a less common side effect, tacrolimus has been documented to cause several potentially life-threatening arrhythmias [62,63,64]. There are several case reports citing QT prolongation and supraventricular arrhythmias in both pediatric and adult transplant patients [62,63,64]. Tacrolimus may also prolong the QT/QTc interval and cause Torsade de Pointes. It should be avoided in cases with congenital long QT syndrome [65]. Periodic electrocardiograms and electrolyte monitoring should be considered during the treatment of patients with congestive heart failure, electrolyte imbalances, bradyarrhythmia, and those on certain antiarrhythmic medications that may cause QT prolongation [66]. In addition, patients should avoid eating grapefruit or drinking grapefruit juice, as it may increase tacrolimus whole blood trough concentrations and elevate the risk of serious adverse reactions, including QT prolongation [67].

Furthermore, there are not only cardiotoxicities associated with tacrolimus use but also metabolic effects. One such effect is dyslipidemia, which is present in 40–66% of liver transplant patients and is a contributing factor in the immunosuppressive role of tacrolimus [68]. With the demonstrated increase in total cholesterol, tacrolimus demonstrates an increase in low-density lipoprotein cholesterol, a decrease in high-density lipoprotein cholesterol, and an increase in triglycerides [69]. In addition, there has been a documented increase in the incidence of diabetes mellitus in post-transplant patients. The risk of post-transplant diabetes mellitus was 53% greater in patients initially treated with tacrolimus after kidney transplantation [70]. African-American and Hispanic kidney transplant recipients are at higher risk [71]. In some cases, new-onset diabetes may be reversible. Blood glucose levels should be closely monitored in patients using tacrolimus, especially given the increased risk of diabetes. Patients with new-onset diabetes post-transplant using tacrolimus were found to be more likely to develop significant hypomagnesemia [72].

Hypomagnesemia has also been found to predict the onset of diabetes mellitus in post-transplant patients with either kidney or liver transplants [58,71,72,73]. Tacrolimus use increases the risk of hyponatremia and hyperkalemia [74]. The malabsorption of magnesium, sodium, and potassium suggests that tacrolimus contributes to renal tubular dysfunction, which may be linked to its nephrotoxic effects [74].

Immunosuppression is necessary in transplant patients to prevent graft-versus-host disease, but it is a careful balancing act, as immunosuppression increases the susceptibility to infection. It has been documented that a higher tacrolimus trough level may indicate a greater immunosuppression level and, thus, increase the risk of infection by rare viruses such as JC virus, BK polyomavirus, and co-infections [75]. Some studies have demonstrated an association between Gram-negative bacilli and pneumonia in patients receiving tacrolimus therapy [76]. There was also an increase in CMV infections among liver transplant recipients [76]. It was also found that the increased risk of infection with pathogens such as Gram-negative bacilli causing pneumonia or CMV infections can be prevented if the patient takes appropriate prophylactic medications [76].

Tacrolimus is commonly used in pediatric transplant patients. Its use is associated with a range of adverse effects that are magnified by the unique physiological characteristics of children. Pediatric patients, especially those younger than 2 years old, often metabolize tacrolimus at faster rates compared to older children or adults, leading to lower weight-adjusted doses but potentially higher exposure to tacrolimus-related toxicities [77]. A retrospective study by Prusinskas et al. found that younger pediatric liver transplant recipients are more likely to be rapid metabolizers, with the tacrolimus concentration-to-dose ratio being a helpful tool in predicting individualized dosing and minimizing complications such as PTLD, especially in the presence of EBV infections [77]. Although severe neurotoxicity is more prevalent in adults than children, mild neurotoxicity, such as myalgias, headaches, and fatigue, is prevalent in younger children [78]. However, these neurotoxic adverse events generally do not require patients to discontinue their tacrolimus regimen. Renal toxicity is also a concern in pediatric patients taking tacrolimus. Children are more vulnerable to dose-dependent renal dysfunction. It is estimated that up to 20% of pediatric liver transplant patients experience hypertension, a well-recognized adverse effect of calcineurin inhibitors, which may be under-recognized due to challenges in accurate blood pressure monitoring in children [79]. The hypertensive effect of tacrolimus contributes to its nephrotoxicity because it tends to be less frequently recognized in children and leads to a progressive decline in renal function. Tacrolimus has also been linked to metabolic complications, like post-transplant diabetes mellitus, with a higher risk observed in older pediatric patients [80]. Genetic factors, such as variants in the POR and ABCB1 genes, have been associated with an increased risk of PTDM, which emphasizes the need for personalized management strategies. Additionally, tacrolimus can lead to other adverse effects in pediatric transplant recipients, including dyslipidemia and food allergies, which are less common in adults [79].

**Table 1:** Overview of tacrolimus-related toxicities in post-transplant patients.

Toxicity Category	Manifestations	Mechanism and Risk Factors	Clinical Management
Nephrotoxicity	Increased serum creatinine, epithelial vacuolization, vasospasm, and interstitial fibrosis [48]	Calcineurin inhibition; high prevalence of FKBP12 in the kidney; associated with high-dose tacrolimus (>20 ng/mL) and CYP3A5 non-expressor genotype [48,50]	Reduce tacrolimus dose; monitor renal function; avoid nephrotoxic co-medications [49]
Neurotoxicity	Altered consciousness, tremors, headaches, posterior reversible encephalopathy syndrome (PRES), optic neuropathy, and psychosis [51,52,53,54]	Calcineurin inhibition affecting the CNS; optic neuropathy linked to structural changes in optic pathways [54]	Discontinue or reduce tacrolimus dose; symptoms like PRES and optic neuropathy are reversible with early intervention [55,57]
Cardiotoxicity	Hypertension, myocardial hypertrophy, QT prolongation, Torsade de Pointes, and arrhythmias (supraventricular and bradyarrhythmias) [60,61,62,63,64,65,66,67]	Vascular effects of calcineurin inhibition; electrolyte imbalances; grapefruit juice interaction increasing drug levels [66,67]	Monitor blood pressure, electrolytes, and ECG; avoid grapefruit products; consider alternatives in high-risk patients [66,67]
Metabolic effects	Dyslipidemia (increased LDL, triglycerides, and decreased HDL), post-transplant diabetes mellitus, hypomagnesemia, hyponatremia, and hyperkalemia [68,69,70,72,74]	Dysregulation of lipid and glucose metabolisms; renal tubular dysfunction leading to electrolyte imbalances [74]	Monitor lipid profile, blood glucose, and electrolytes; manage dyslipidemia and diabetes mellitus with appropriate medications [69,71]
Infectious susceptibility	Increased risk of infections (e.g., JC virus, BK polyomavirus, Gram-negative pneumonia, and CMV infections) [75,76]	High tacrolimus trough levels leading to overimmunosuppression; increased susceptibility to rare and opportunistic infections [75]	Use prophylactic antimicrobials; monitor for infection markers; optimize tacrolimus dose to balance immunosuppression and infection risk [75]

## 5. Mycophenolate-Related Toxicity in Post-Transplant Patients

Mycophenolate mofetil is an immunosuppressive drug used to prevent organ rejection in post-transplant patients [20,81,82]. While its efficacy in immunosuppression is well established, the drug is not without adverse effects (Table 2). In addition to its teratogenic effects, which increase the risk of congenital malformations and pregnancy loss, mycophenolate is associated with a range of toxicities that can affect multiple organ systems [83,84,85]. These include gastrointestinal tract effects, hematologic system effects, immune responses to infection, and the development of malignancies [19,86,87,88].

Gastrointestinal side effects are some of the most common complications associated with mycophenolate therapy occurring in up to 45% of patients [89,90]. Mycophenolate acts by inhibiting IMP dehydrogenase, a key enzyme in the synthesis of guanine nucleotides [18]. This action suppresses immune function by slowing the proliferation of lymphocytes, but it also affects the rapidly dividing cells of the GI tract, leading to villous atrophy. Additionally, the drug is metabolized by enterocytes, producing toxic metabolites that can directly damage the gastrointestinal mucosa leading to erosive enterocolitis. This action is exacerbated by enterohepatic recirculation, where the drug is glucuronidated in the liver to form MPAG. MPAG is then excreted into the bile and released into the intestines, where the bacterial enzyme β-glucuronidase converts it back into the active form, which can be reabsorbed increasing the duration of exposure of the gut to the drug [91].

Diarrhea is the most commonly reported gastrointestinal side effect of mycophenolate, accounting for 50 to 90% of related toxicities [89]. This is attributed to enterocyte dependency on purine synthesis, leading to defective growth and the replication of small bowel epithelial cells, causing the disruption of fluid absorption [90]. The severity of the diarrhea can range from mild, manageable with dose adjustments, to severe, requiring discontinuation of therapy [92]. The diarrhea is typically dose dependent and concentration dependent, often presenting as high-volume stools accompanied by abdominal pain or cramping [93]. If the upper gastrointestinal tract is involved, patients might also develop nausea (29%), vomiting (23%), and anorexia [90]. These symptoms may mimic other causes of GI distress, making it crucial for clinicians to differentiate between drug-related side effects and other possible causes such as inflammatory etiologies, infections, or graft-versus-host disease [94].

It has been reported that up to 45% of patients experience gastrointestinal complications following mycophenolate therapy [89,90]. This can impair clinical outcomes, because many of these complications can lead to sub-therapeutic dosages of mycophenolate or its discontinuation [95]. This led to the development of an alternative formulation of mycophenolate that could reduce these complications, called enteric-coated mycophenolate sodium (EC-MPS). EC-MPS is a delayed-release medication that works by avoiding the release of mycophenolate into the stomach and instead releases in the small intestines, which has a neutral pH [96]. Compared to the traditional mycophenolate, EC-MPS has been found to significantly reduce gastrointestinal complications, improve well-being, and increase the maximum tolerated dose of mycophenolate [97,98,99]. Additionally, there was minimal difference in efficacy between mycophenolate and EC-MPS, which demonstrates that EC-MPS is a viable alternative to mycophenolate in patients struggling with gastrointestinal complications [100,101].

Mycophenolate therapy can also induce hematologic toxicities, primarily affecting the bone marrow’s ability to produce blood cells. This can manifest as anemia, leukopenia, or thrombocytopenia, occurring in 15–60%, 10–45%, and 8–14% of cases, respectively [88]. Patients may experience fatigue, weakness, and pallor due to anemia, while leukopenia can lead to increased susceptibility to infection, and thrombocytopenia may present as bruising, petechia, and mucosal bleeding [102,103,104]. These effects are mostly mild in nature, dependent on the concentration of the drug, and reversible after dose reduction or pharmacotherapy modifications [88]. Differences in the incidence of hematological toxicity may be attributable to the degree of myelosuppression, genetic predisposition, and the composition of the multi-drug regimen [45,105,106].

Anemia is the most common of the hematological toxicities and is particularly important, as it has been associated with increased mortality and a higher risk of graft failure in transplant recipients [107]. In kidney transplant patients, anemia is further complicated by reduced erythropoietin production due to progressive renal disease and decreased graft excretory function, as well as a high prevalence of iron deficiency. Thus, it becomes difficult to investigate whether the adverse effect can be solely attributable to mycophenolate therapy [108,109].

Leukopenia is one of the most significant concerns with mycophenolate therapy, as it suppresses lymphocyte proliferation. While this suppression is desirable to prevent transplant rejection, it compromises the body’s ability to mount an immune response, increasing the risk of infection [19]. Infections are the leading cause of mortality among cases in the early post-transplant period, and the immunosuppressive effects of mycophenolate render these patients vulnerable to both common infections and opportunistic pathogens [110]. CMV is the most frequently associated infection with mycophenolate occurring in a dose-dependent fashion, leading to various complications including colitis, meningoencephalitis, pneumonitis, and retinitis [111,112,113,114]. Other associated viral infections include EBV, which can result in PTLD; Herpes simplex virus, which may lead to herpes simplex encephalitis; BK virus, which is associated with nephropathy and viremia; and Varicella-zoster virus, which can cause shingles and disseminated disease [115,116,117,118]. Invasive fungal infections, including aspergillosis and candidiasis, are also commonly seen and present a significant risk. Aspergillosis can cause pneumonitis, while candidiasis may lead to esophagitis, with both conditions increasing the risk of invasive and disseminated disease when associated with mycophenolate use [119,120,121,122].

Additionally, the immunosuppressive effects of mycophenolate have been shown to increase the risk of malignancies, primarily by reducing the body’s immune surveillance capabilities [123]. Through inhibited lymphocyte proliferation, abnormal cell lines can evade detection and proliferate unchecked [124]. The diminished immune response not only weakens the body’s defense against carcinogens but also impairs its ability to repair UV-damaged cells and eliminate oncogenic viruses [125,126,127]. As a result, there is an increased susceptibility to malignancies such as non-melanoma skin cancer, specifically squamous cell carcinoma and PTLD [47,125,128]. PTLD is a spectrum of lymphoid proliferative conditions that range from benign hyperplasia to aggressive lymphomas, with most cases being associated with EBV infection. Mycophenolate’s immunosuppressive effects allow EBV-infected B cells to proliferate unchecked, increasing the risk of development and progression. This risk is further amplified in patients who are EBV seronegative at the time of transplant, as they are more likely to develop primary EBV infection post-transplant [129]. Balancing the benefits of immunosuppression with the potential of serious complications, especially in relation to the duration and dosage of mycophenolate therapy, is essential for optimizing patient outcomes [130].

Mycophenolate is widely used in pediatric transplant patients as part of immunosuppressive regimens, but it is associated with several adverse events. One of the most common and concerning side effects is hematologic toxicity, particularly leukopenia, which has been reported in a significant proportion of pediatric patients. A retrospective study by Varnell et al. found that 24% of pediatric kidney transplant recipients developed mycophenolate-related leukopenia, which typically improved after dose adjustments or discontinuation of the drug [106]. Genetic variants, such as UGT2B7-900A>G and SNPs in IMPDH1, were also found to influence the risk and timing of leukopenia. This suggests that genetic testing may help predict which children are more susceptible to this side effect. Gastrointestinal intolerance is also a common adverse effect caused by mycophenolate usage. A retrospective review by Ohmann et al. found that 35.6% of pediatric patients experienced gastrointestinal intolerance to mycophenolate, which led to dose holding or discontinuation, with vomiting, nausea, diarrhea, and abdominal pain being the most common symptoms [131]. Infections are another significant risk associated with mycophenolate treatment, with CMV being the most frequently reported infection in pediatric transplant recipients [132]. While CMV infections are more commonly observed in adult patients, they are less common in pediatric cases, and no significant association between mycophenolate and infections has been found in the pediatric population.

**Table 2:** Overview of mycophenolate-related toxicities in post-transplant patients.

Toxicity Category	Manifestations	Mechanism and Risk Factors	Clinical Management
Gastrointestinal effects	Diarrhea (50–90% of GI toxicities), nausea, vomiting, anorexia, abdominal pain, and erosive enterocolitis [89,90,93]	Inhibition of IMP dehydrogenase affecting rapidly dividing GI cells; toxic metabolites from enterohepatic recirculation increase gut exposure [18,91]	Dose adjustments; differentiate from other GI causes (e.g., infections and GVHD); supportive therapy [93,94]
Hematological effects	Anemia (15–60%), leukopenia (10–45%), and thrombocytopenia (8–14%); fatigue, pallor, increased infection risk, bruising, and petechiae [87,102,103,104,107]	Myelosuppression reduces blood cell production; anemia may also relate to renal dysfunction and iron deficiency in transplant patients [105,107]	Monitor blood counts regularly; dose reduction or pharmacotherapy modification; address underlying anemia causes [87,107]
Infectious susceptibility	CMV (colitis, pneumonitis, and retinitis), EBV (post-transplant lymphoproliferative disorder, PTLD), BK virus (nephropathy), and fungal infections (aspergillosis and candidiasis) [111,112,113,114,115,116,117,118,119,120,121,122]	Suppressed lymphocyte proliferation impairs immune response; dose-dependent infection risk; opportunistic infections increase due to reduced immune surveillance [19,110]	Prophylactic antivirals and antifungals; monitor for infection markers; adjust immunosuppressive therapy as needed [110]
Malignancy susceptibility	Non-melanoma skin cancers (e.g., squamous cell carcinoma) and PTLD (EBV-associated lymphomas) [47,125,126,127,128,129]	Impaired immune surveillance reduces ability to detect and repair abnormal cells; EBV-infected B cells proliferate unchecked, especially in EBV-seronegative patients at transplant [123,124,129]	Regular skin checks; minimize immunosuppression when possible; consider EBV monitoring and prophylaxis [130]
Teratogenic effects	Increased risk of congenital malformations and pregnancy loss [85]	Mycophenolate crosses the placenta and affects rapidly dividing fetal cells [84]	Avoid in pregnancy; use effective contraception for patients of childbearing potential; switch to safer agents [83]

## 6. Drug–Drug Interactions and Risk Factors for Enhanced Toxicity in Post-Transplant Patients

Tacrolimus has a narrow therapeutic index, with a high risk of toxicity if serum levels become elevated. Its metabolism and, therefore, its bioavailability is highly sensitive to drug interactions, particularly with medications that inhibit the CYP3A4 and CYP3A5 enzymes, which play key roles in tacrolimus metabolism in the liver and small intestine [133]. Established drug interactions with tacrolimus include those with calcium channel blockers (CCBs), azole antifungals, macrolide antibiotics, protease inhibitors, amiodarone, and certain selective serotonin reuptake inhibitors (SSRIs). Given the high prevalence of hypertension in renal transplant patients (70–85%), CCBs are commonly co-administered but can inhibit CYP3A enzymes, potentially leading to toxic tacrolimus accumulation [134]. Azole antifungals, like fluconazole, are potent CYP3A inhibitors and have been shown to cause toxic blood levels of tacrolimus in liver transplant patients, even with dose reductions of over 50% [135]. Macrolide antibiotics, such as clarithromycin and erythromycin, similarly inhibit CYP3A, slowing tacrolimus metabolism and increasing the risk of toxic buildup and hospitalization [136]. Protease inhibitors, which also inhibit CYP3A and P-glycoprotein, further prolong tacrolimus’s half-life, requiring careful dose adjustments to avoid toxicity [137]. Amiodarone, metabolized through CYP3A, has been linked to increased mortality and graft dysfunction when combined with tacrolimus in transplant patients [138]. Additionally, adverse events have been associated after cardiac and liver transplants in patients using both tacrolimus and sirolimus [139,140]. Liver transplant patients experienced higher rates of hepatic artery thrombosis, sepsis, graft loss, and death when using both sirolimus and tacrolimus compared to tacrolimus alone [140]. Increased rates of renal dysfunction and impaired wound healing were found in patients using sirolimus and tacrolimus after cardiac transplants [139]. Finally, SSRIs like nefazodone, which also rely on CYP3A metabolism, can result in toxic tacrolimus levels unless dosing adjustments are made [141].

Mycophenolate, primarily metabolized by UDP-glucuronosyltransferase, has a narrow therapeutic window, making it susceptible to toxicity or reduced efficacy if its absorption and metabolism are altered by drug interactions. Proton pump inhibitors, antacids, and bile acid sequestrants are known to interfere with mycophenolate’s gastrointestinal absorption, reducing its bioavailability and potentially increasing the risk of transplant rejection [142,143,144]. Additionally, acyclovir and ganciclovir can elevate the serum levels of both the antiviral agents and mycophenolate by competitively inhibiting mycophenolate’s renal excretion [145,146]. Furthermore, antibiotics such as metronidazole and ciprofloxacin can disrupt gut flora, decreasing mycophenolate absorption and leading to lower serum levels [147,148].

Tacrolimus and mycophenolate are commonly co-administered in transplant immunosuppression regimens, but their combined use can present risks of toxicity or reduced efficacy due to overlapping metabolic pathways and interactions [144,149]. Both drugs rely on UDP-glucuronosyltransferase, cytochrome P450 enzymes, and P-glycoprotein for metabolism, with renal excretion as a primary route. Studies indicate that co-administration can lead to elevated mycophenolate levels and reduced tacrolimus bioavailability, potentially resulting in myelosuppressive side effects like anemia and leukopenia, or, in severe cases, transplant rejection [150]. Alternatively, if tacrolimus reaches toxic levels, nephrotoxicity and neurotoxicity may occur. However, many patients tolerate this combination well within the therapeutic range, experiencing only mild to moderate side effects such as diarrhea, headache, dyspepsia, and vomiting. To ensure effective immunosuppression and minimize risks, regular monitoring of plasma concentrations of both agents is essential [151,152,153].

When assessing the toxicity risks of mycophenolate and tacrolimus, it is essential to consider additional patient-specific factors. Genetic polymorphisms affecting key transport and metabolizing enzymes such as CYP3A4, CYP3A5, P-glycoprotein, and UDP-glucuronosyltransferase can significantly impact the bioavailability and clearance of these immunosuppressants, potentially leading to toxicity [154,155,156]. Age is also a notable risk factor; older patients often have reduced hepatic and renal functions, which can result in drug accumulation and toxicity, while younger patients may need higher doses to achieve therapeutic levels [157]. Similarly, any patient with renal or hepatic impairment faces an elevated risk of toxic drug accumulation. For these individuals, close monitoring of blood levels is necessary to ensure the dosages are carefully adjusted within the therapeutic range [158,159].

## 7. Strategies to Mitigate Post-Transplant Toxicity

The use of tacrolimus and mycophenolate in post-transplant immunosuppression is essential for preventing transplant rejection but is associated with significant risks of toxicity. Strategies to mitigate these adverse effects include monitoring protocols for early detection, personalized therapeutic adjustments, and using pharmacogenomics.

Effective monitoring protocols for tacrolimus and mycophenolate focus on maintaining target drug levels to prevent toxicity and rejection. For tacrolimus, achieving trough levels of 7–12 ng/mL early post-transplant (at the first month) reduces the risk of acute rejection (AR) by 86% compared to trough levels of 4–7 ng/mL, while levels between 5.35 and 7.15 ng/mL manage to balance AR prevention and infection risk [160,161]. For mycophenolate, strategies like pharmacokinetic monitoring and target concentration intervention help ensure appropriate exposure [162]. Insufficient levels can lead to rejection, while excessive levels increase the risk of toxicities, including anemia, leucopenia, and diarrhea [163].

These protocols highlight the importance of personalized therapeutic drug monitoring (TDM) for optimal transplant outcomes [164]. Newer methods like dried blood spot (DBS) sampling and immunobiograms have shown promise for accurate TDM by improving efficiency and predicting patient sensitivity to immunosuppressants, simplifying outpatient management [164,165]. For tacrolimus, area-under-the-concentration (AUC)-based monitoring using capillary sampling with pharmacokinetic model-derived Bayesian estimators was found to be an accurate and precise measure of exposure that can be conducted at the patient’s home [166]. Similarly, limited sampling strategies have been developed for MPA to estimate the AUC, aiding in precise dose adjustments [167]. Alternative approaches, such as intracellular monitoring or assessing calcineurin pathway activity (e.g., NFATc1 amplification), may correlate with better clinical outcomes but require further validation [168,169].

Pharmacogenomics offers an emerging avenue for mitigating drug toxicity by tailoring immunosuppressive therapy based on genetic predispositions. Variations in the CYP3A5 gene significantly influence tacrolimus metabolism, where CYP3A5 expressers exhibit a 1.48-fold higher clearance rate compared to non-expressers, which demonstrates the need for genotype-guided dosing adjustments to improve therapeutic outcomes [170]. However, pharmacogenetic variability appears to have a limited effect on the metabolism of MPA, potentially due to low allele frequencies. The efficacy of mycophenolate has been linked to its action on IMP dehydrogenase. A post-transplant study showed that MPA strongly inhibits IMP dehydrogenase activity in stimulated peripheral blood mononuclear cells (PBMCs), and it found that enzyme capacity was significantly reduced after dosing [171]. Moreover, a lower baseline IMP dehydrogenase capacity in non-stimulated PBMCs early post-transplant was associated with dose adjustments, which demonstrated the potential of pharmacogenomics to optimize mycophenolate therapy based on individual enzyme responses.

## 8. Future Directions and Emerging Therapies

As research into immunosuppression evolves, new strategies and therapies are being developed to minimize the toxicity of tacrolimus and mycophenolate, aiming to improve patient outcomes and reduce the burden of adverse effects. For post-transplant patients, individualized treatment strategies for tacrolimus and mycophenolate are essential to minimize toxicity and optimize clinical outcomes. Given the high variability in tacrolimus pharmacokinetics, TDM is critical, but dosing algorithms, which utilize factors such as CYP3A genotype and hematocrit, are increasingly used to refine dosing [172]. While some algorithms perform better than weight-based dosing in terms of achieving target concentrations more efficiently, their clinical benefit in reducing tacrolimus-related toxicity remains unclear, highlighting the need for external validation and the consideration of additional biomarkers (e.g., unbound plasma tacrolimus levels) for more accurate monitoring [173]. Although no dosing algorithms have been found for mycophenolate, the future of individualized treatment for its therapy relies on integrating pharmacogenetic, pharmacokinetic, and pharmacodynamic approaches to optimize patient outcomes [173,174]. By monitoring IMP dehydrogenase enzyme activity in PBMCs, clinicians can better assess the biological response to MPA, offering a more personalized approach compared to traditional drug level monitoring [173]. Although practical implementation remains challenging, these advanced strategies hold promise for reducing transplant rejection and improving long-term graft survival.

Alternative treatments for post-transplantation toxicity may involve switching immunosuppressive agents based on specific patient needs. For instance, replacing MPA with everolimus in patients with neutropenia has shown to be a safe and effective strategy, with a reduction in neutropenia episodes and stable renal function over the long term [175]. Additionally, sirolimus has been proposed as a substitute for tacrolimus in various transplant settings, with studies indicating comparable or even improved outcomes in terms of survival and chronic rejection, particularly in lung transplant recipients [176,177]. However, while newer alternatives like belatacept may offer some benefits, such as a reduced incidence of new-onset diabetes, they are associated with a higher risk of acute rejection and worse allograft survival compared to tacrolimus [178]. Careful consideration of individual patient factors, including rejection risk and long-term outcomes, is essential for optimizing and selecting immunosuppressive therapy.

## 9. Conclusions

Tacrolimus and mycophenolate are essential immunosuppressive agents for preventing organ rejection in post-transplant patients, but their use is associated with significant toxicity that requires careful management. Tacrolimus can cause nephrotoxicity, neurotoxicity, and metabolic disturbances such as diabetes mellitus and dyslipidemia. Its nephrotoxic effects, which are often dose-dependent, may lead to chronic kidney injury, while its neurotoxic effects range from tremors and headaches to severe conditions like posterior reversible encephalopathy syndrome. Cardiovascular risks, including hypertension and arrhythmias, further complicate its use. Similarly, mycophenolate is associated with gastrointestinal toxicity, manifesting as diarrhea, nausea, and, in severe cases, erosive colitis. It also poses risks of hematologic complications like anemia and leukopenia, which increase infection susceptibility and compromise immune function.

Emerging strategies, including TDM and pharmacogenomics, offer valuable tools for mitigating these adverse effects. For tacrolimus, maintaining optimal trough levels through TDM can help minimize nephrotoxicity and other dose-related toxicities. Genetic insights, such as identifying CYP3A5 polymorphisms, allow for individualized dosing to reduce variability in drug metabolism and exposure. For mycophenolate, monitoring IMP dehydrogenase activity provides a pharmacodynamic measure that can guide dose adjustments, potentially reducing gastrointestinal and hematologic side effects.

While alternative newer immunosuppressants, like sirolimus, everolimus, and belatacept, and more traditional immunosuppressants, like azathioprine and cyclosporine, can be options for patients who cannot tolerate these drugs, they come with their own risks and limitations. Moving forward, integrating advanced monitoring techniques with personalized medicine will be crucial to balancing efficacy and safety, reducing the burden of side effects, and improving patient outcomes in post-transplant care.
